# Supplementary material for: Modeling glioblastoma heterogeneity as a dynamic network of cell states
Source: Mol Syst Biol. 2021 Sep 16;17(9):e10105. doi: 10.15252/msb.202010105 (PMC8444284; doi:10.15252/msb.202010105)
Supplement: Supplementary file 5 — Source Data for Figure 3 [file MSB-17-e10105-s001.zip › Figure3A_sourcedata/GSEA_3065/hallmarks_state6.GseaPreranked.1623416308702/HALLMARK_E2F_TARGETS.html]

Details for gene set HALLMARK\_E2F\_TARGETS[GSEA]

|  || Dataset | state6 |
| Phenotype | NoPhenotypeAvailable |
| Upregulated in class | na\_neg |
| GeneSet | HALLMARK\_E2F\_TARGETS |
| Enrichment Score (ES) | -0.4731595 |
| Normalized Enrichment Score (NES) | -2.0714602 |
| Nominal p-value | 0.0 |
| FDR q-value | 0.0 |
| FWER p-Value | 0.0 |
Table: GSEA Results Summary

  

Fig 1: Enrichment plot: HALLMARK\_E2F\_TARGETS      
 Profile of the Running ES Score & Positions of GeneSet Members on the Rank Ordered List

  

| PROBE | GENE SYMBOL | GENE\_TITLE | RANK IN GENE LIST | RANK METRIC SCORE | RUNNING ES | CORE ENRICHMENT || 1 | CDKN1A |  |  | 1 | 0.477 | 0.0347 | No |
| 2 | TP53 |  |  | 116 | 0.205 | 0.0365 | No |
| 3 | PPM1D |  |  | 386 | 0.139 | 0.0154 | No |
| 4 | GSPT1 |  |  | 455 | 0.127 | 0.0168 | No |
| 5 | MTHFD2 |  |  | 926 | 0.090 | -0.0312 | No |
| 6 | EZH2 |  |  | 940 | 0.089 | -0.0262 | No |
| 7 | MYBL2 |  |  | 991 | 0.086 | -0.0257 | No |
| 8 | SMC6 |  |  | 1023 | 0.084 | -0.0231 | No |
| 9 | SSRP1 |  |  | 1124 | 0.080 | -0.0289 | No |
| 10 | NOLC1 |  |  | 1181 | 0.076 | -0.0298 | No |
| 11 | PSIP1 |  |  | 1372 | 0.068 | -0.0469 | No |
| 12 | CDK4 |  |  | 1490 | 0.064 | -0.0558 | No |
| 13 | DEK |  |  | 1535 | 0.063 | -0.0563 | No |
| 14 | TIMELESS |  |  | 1584 | 0.061 | -0.0574 | No |
| 15 | POLD2 |  |  | 1589 | 0.061 | -0.0534 | No |
| 16 | STMN1 |  |  | 1649 | 0.058 | -0.0560 | No |
| 17 | USP1 |  |  | 1712 | 0.056 | -0.0591 | No |
| 18 | NBN |  |  | 1820 | 0.053 | -0.0676 | No |
| 19 | MYC |  |  | 2081 | 0.045 | -0.0945 | No |
| 20 | JPT1 |  |  | 2094 | 0.045 | -0.0926 | No |
| 21 | NAP1L1 |  |  | 2171 | 0.043 | -0.0983 | No |
| 22 | CBX5 |  |  | 2269 | 0.039 | -0.1067 | No |
| 23 | PTTG1 |  |  | 2304 | 0.038 | -0.1078 | No |
| 24 | MCM6 |  |  | 2484 | 0.034 | -0.1261 | No |
| 25 | RPA1 |  |  | 2511 | 0.033 | -0.1268 | No |
| 26 | RPA3 |  |  | 2563 | 0.032 | -0.1304 | No |
| 27 | ANP32E |  |  | 2570 | 0.032 | -0.1287 | No |
| 28 | PHF5A |  |  | 2732 | 0.028 | -0.1454 | No |
| 29 | CDC25A |  |  | 2768 | 0.027 | -0.1474 | No |
| 30 | CCP110 |  |  | 2781 | 0.027 | -0.1469 | No |
| 31 | TMPO |  |  | 2978 | 0.022 | -0.1680 | No |
| 32 | CKS1B |  |  | 2985 | 0.022 | -0.1670 | No |
| 33 | PLK1 |  |  | 3116 | 0.020 | -0.1807 | No |
| 34 | AK2 |  |  | 3141 | 0.019 | -0.1821 | No |
| 35 | MRE11 |  |  | 3289 | 0.016 | -0.1980 | No |
| 36 | HNRNPD |  |  | 3359 | 0.014 | -0.2050 | No |
| 37 | HMGB3 |  |  | 3377 | 0.014 | -0.2060 | No |
| 38 | RACGAP1 |  |  | 3432 | 0.012 | -0.2114 | No |
| 39 | DUT |  |  | 3596 | 0.009 | -0.2296 | No |
| 40 | RFC2 |  |  | 3618 | 0.009 | -0.2314 | No |
| 41 | RAD51C |  |  | 3629 | 0.009 | -0.2319 | No |
| 42 | SLBP |  |  | 3741 | 0.006 | -0.2444 | No |
| 43 | DCTPP1 |  |  | 3814 | 0.004 | -0.2524 | No |
| 44 | CDC25B |  |  | 3887 | 0.003 | -0.2606 | No |
| 45 | UBR7 |  |  | 3892 | 0.003 | -0.2609 | No |
| 46 | SHMT1 |  |  | 3901 | 0.002 | -0.2616 | No |
| 47 | CDC20 |  |  | 3996 | 0.001 | -0.2725 | No |
| 48 | POLE4 |  |  | 4140 | -0.002 | -0.2889 | No |
| 49 | RAD1 |  |  | 4196 | -0.003 | -0.2951 | No |
| 50 | MSH2 |  |  | 4317 | -0.005 | -0.3086 | No |
| 51 | SNRPB |  |  | 4387 | -0.007 | -0.3162 | No |
| 52 | MCM7 |  |  | 4435 | -0.008 | -0.3211 | No |
| 53 | CDKN1B |  |  | 4475 | -0.008 | -0.3250 | No |
| 54 | HMGA1 |  |  | 4523 | -0.009 | -0.3298 | No |
| 55 | HUS1 |  |  | 4532 | -0.009 | -0.3300 | No |
| 56 | CNOT9 |  |  | 4622 | -0.011 | -0.3395 | No |
| 57 | POLE |  |  | 4643 | -0.012 | -0.3410 | No |
| 58 | CCNB2 |  |  | 4736 | -0.013 | -0.3507 | No |
| 59 | PPP1R8 |  |  | 4798 | -0.014 | -0.3568 | No |
| 60 | DDX39A |  |  | 4809 | -0.014 | -0.3569 | No |
| 61 | ZW10 |  |  | 4841 | -0.015 | -0.3594 | No |
| 62 | ASF1A |  |  | 4888 | -0.016 | -0.3635 | No |
| 63 | CCNE1 |  |  | 4890 | -0.016 | -0.3624 | No |
| 64 | ASF1B |  |  | 4895 | -0.016 | -0.3617 | No |
| 65 | CENPM |  |  | 5089 | -0.020 | -0.3826 | No |
| 66 | DCK |  |  | 5091 | -0.020 | -0.3813 | No |
| 67 | POLA2 |  |  | 5164 | -0.022 | -0.3881 | No |
| 68 | CTCF |  |  | 5167 | -0.022 | -0.3867 | No |
| 69 | NUP153 |  |  | 5222 | -0.023 | -0.3913 | No |
| 70 | IPO7 |  |  | 5324 | -0.025 | -0.4012 | No |
| 71 | PRPS1 |  |  | 5347 | -0.025 | -0.4020 | No |
| 72 | PDS5B |  |  | 5359 | -0.025 | -0.4014 | No |
| 73 | ORC2 |  |  | 5449 | -0.027 | -0.4097 | No |
| 74 | PMS2 |  |  | 5458 | -0.028 | -0.4086 | No |
| 75 | NME1 |  |  | 5479 | -0.028 | -0.4089 | No |
| 76 | CTPS1 |  |  | 5522 | -0.029 | -0.4116 | No |
| 77 | CDCA8 |  |  | 5535 | -0.029 | -0.4109 | No |
| 78 | STAG1 |  |  | 5538 | -0.029 | -0.4090 | No |
| 79 | LMNB1 |  |  | 5545 | -0.029 | -0.4075 | No |
| 80 | GINS1 |  |  | 5565 | -0.030 | -0.4076 | No |
| 81 | RBBP7 |  |  | 5605 | -0.031 | -0.4099 | No |
| 82 | NAA38 |  |  | 5848 | -0.035 | -0.4354 | No |
| 83 | EIF2S1 |  |  | 5897 | -0.036 | -0.4383 | No |
| 84 | RFC3 |  |  | 6078 | -0.040 | -0.4563 | No |
| 85 | TUBG1 |  |  | 6104 | -0.040 | -0.4563 | No |
| 86 | SPC24 |  |  | 6135 | -0.041 | -0.4567 | No |
| 87 | SPAG5 |  |  | 6146 | -0.041 | -0.4549 | No |
| 88 | AURKB |  |  | 6215 | -0.043 | -0.4596 | No |
| 89 | SRSF2 |  |  | 6250 | -0.044 | -0.4604 | No |
| 90 | BARD1 |  |  | 6284 | -0.044 | -0.4610 | No |
| 91 | CDKN3 |  |  | 6291 | -0.044 | -0.4584 | No |
| 92 | TRA2B |  |  | 6303 | -0.045 | -0.4565 | No |
| 93 | RANBP1 |  |  | 6309 | -0.045 | -0.4538 | No |
| 94 | ING3 |  |  | 6389 | -0.047 | -0.4595 | No |
| 95 | RPA2 |  |  | 6501 | -0.050 | -0.4688 | No |
| 96 | TFRC |  |  | 6511 | -0.050 | -0.4662 | No |
| 97 | XRCC6 |  |  | 6553 | -0.052 | -0.4671 | No |
| 98 | SYNCRIP |  |  | 6600 | -0.053 | -0.4686 | No |
| 99 | HMMR |  |  | 6640 | -0.054 | -0.4692 | Yes |
| 100 | SRSF1 |  |  | 6655 | -0.054 | -0.4669 | Yes |
| 101 | TBRG4 |  |  | 6657 | -0.054 | -0.4630 | Yes |
| 102 | PRIM2 |  |  | 6678 | -0.055 | -0.4613 | Yes |
| 103 | MLH1 |  |  | 6702 | -0.056 | -0.4599 | Yes |
| 104 | BUB1B |  |  | 6717 | -0.056 | -0.4574 | Yes |
| 105 | PSMC3IP |  |  | 6749 | -0.057 | -0.4568 | Yes |
| 106 | MKI67 |  |  | 6750 | -0.057 | -0.4526 | Yes |
| 107 | MCM5 |  |  | 6765 | -0.058 | -0.4500 | Yes |
| 108 | MXD3 |  |  | 6808 | -0.059 | -0.4506 | Yes |
| 109 | TUBB |  |  | 6820 | -0.059 | -0.4475 | Yes |
| 110 | TCF19 |  |  | 6859 | -0.061 | -0.4475 | Yes |
| 111 | CIT |  |  | 6948 | -0.063 | -0.4531 | Yes |
| 112 | RAD21 |  |  | 6984 | -0.064 | -0.4525 | Yes |
| 113 | TACC3 |  |  | 7022 | -0.066 | -0.4520 | Yes |
| 114 | BIRC5 |  |  | 7024 | -0.066 | -0.4473 | Yes |
| 115 | SMC3 |  |  | 7087 | -0.067 | -0.4495 | Yes |
| 116 | KIF22 |  |  | 7098 | -0.068 | -0.4457 | Yes |
| 117 | KIF18B |  |  | 7103 | -0.068 | -0.4412 | Yes |
| 118 | DONSON |  |  | 7121 | -0.068 | -0.4382 | Yes |
| 119 | PA2G4 |  |  | 7192 | -0.071 | -0.4412 | Yes |
| 120 | RAN |  |  | 7221 | -0.072 | -0.4392 | Yes |
| 121 | RAD51AP1 |  |  | 7236 | -0.072 | -0.4355 | Yes |
| 122 | UNG |  |  | 7249 | -0.073 | -0.4316 | Yes |
| 123 | CSE1L |  |  | 7262 | -0.073 | -0.4276 | Yes |
| 124 | TRIP13 |  |  | 7291 | -0.074 | -0.4255 | Yes |
| 125 | LYAR |  |  | 7341 | -0.076 | -0.4256 | Yes |
| 126 | TOP2A |  |  | 7430 | -0.079 | -0.4301 | Yes |
| 127 | TK1 |  |  | 7499 | -0.081 | -0.4320 | Yes |
| 128 | SPC25 |  |  | 7522 | -0.082 | -0.4285 | Yes |
| 129 | NUDT21 |  |  | 7524 | -0.082 | -0.4226 | Yes |
| 130 | MCM3 |  |  | 7597 | -0.086 | -0.4247 | Yes |
| 131 | CDCA3 |  |  | 7631 | -0.087 | -0.4222 | Yes |
| 132 | DEPDC1 |  |  | 7637 | -0.087 | -0.4164 | Yes |
| 133 | EXOSC8 |  |  | 7671 | -0.089 | -0.4137 | Yes |
| 134 | WDR90 |  |  | 7690 | -0.091 | -0.4091 | Yes |
| 135 | AURKA |  |  | 7700 | -0.091 | -0.4035 | Yes |
| 136 | CDKN2C |  |  | 7743 | -0.093 | -0.4016 | Yes |
| 137 | BRCA2 |  |  | 7772 | -0.094 | -0.3980 | Yes |
| 138 | UBE2S |  |  | 7818 | -0.097 | -0.3961 | Yes |
| 139 | CHEK1 |  |  | 7852 | -0.099 | -0.3927 | Yes |
| 140 | MCM4 |  |  | 7900 | -0.101 | -0.3908 | Yes |
| 141 | TIPIN |  |  | 7921 | -0.102 | -0.3856 | Yes |
| 142 | NUP107 |  |  | 7942 | -0.103 | -0.3804 | Yes |
| 143 | DSCC1 |  |  | 7950 | -0.104 | -0.3736 | Yes |
| 144 | LIG1 |  |  | 7987 | -0.106 | -0.3700 | Yes |
| 145 | RRM2 |  |  | 8001 | -0.107 | -0.3637 | Yes |
| 146 | POLD3 |  |  | 8033 | -0.109 | -0.3593 | Yes |
| 147 | KPNA2 |  |  | 8045 | -0.110 | -0.3526 | Yes |
| 148 | SMC1A |  |  | 8047 | -0.110 | -0.3446 | Yes |
| 149 | WEE1 |  |  | 8054 | -0.111 | -0.3372 | Yes |
| 150 | PCNA |  |  | 8061 | -0.111 | -0.3298 | Yes |
| 151 | CKS2 |  |  | 8077 | -0.112 | -0.3234 | Yes |
| 152 | PRKDC |  |  | 8083 | -0.113 | -0.3157 | Yes |
| 153 | MCM2 |  |  | 8099 | -0.114 | -0.3091 | Yes |
| 154 | POP7 |  |  | 8147 | -0.117 | -0.3060 | Yes |
| 155 | RFC1 |  |  | 8205 | -0.122 | -0.3037 | Yes |
| 156 | XPO1 |  |  | 8216 | -0.123 | -0.2959 | Yes |
| 157 | MMS22L |  |  | 8222 | -0.124 | -0.2875 | Yes |
| 158 | LBR |  |  | 8242 | -0.125 | -0.2805 | Yes |
| 159 | GINS4 |  |  | 8292 | -0.131 | -0.2766 | Yes |
| 160 | KIF4A |  |  | 8300 | -0.132 | -0.2677 | Yes |
| 161 | DIAPH3 |  |  | 8315 | -0.134 | -0.2595 | Yes |
| 162 | NASP |  |  | 8318 | -0.134 | -0.2500 | Yes |
| 163 | KIF2C |  |  | 8328 | -0.135 | -0.2411 | Yes |
| 164 | EED |  |  | 8338 | -0.137 | -0.2322 | Yes |
| 165 | CDK1 |  |  | 8351 | -0.139 | -0.2234 | Yes |
| 166 | ATAD2 |  |  | 8359 | -0.141 | -0.2139 | Yes |
| 167 | NCAPD2 |  |  | 8367 | -0.142 | -0.2043 | Yes |
| 168 | NUP205 |  |  | 8386 | -0.145 | -0.1958 | Yes |
| 169 | HMGB2 |  |  | 8388 | -0.145 | -0.1854 | Yes |
| 170 | MAD2L1 |  |  | 8427 | -0.150 | -0.1788 | Yes |
| 171 | ILF3 |  |  | 8430 | -0.150 | -0.1681 | Yes |
| 172 | MELK |  |  | 8437 | -0.150 | -0.1578 | Yes |
| 173 | DLGAP5 |  |  | 8447 | -0.152 | -0.1477 | Yes |
| 174 | ORC6 |  |  | 8482 | -0.157 | -0.1402 | Yes |
| 175 | UBE2T |  |  | 8489 | -0.159 | -0.1293 | Yes |
| 176 | PNN |  |  | 8524 | -0.167 | -0.1210 | Yes |
| 177 | DNMT1 |  |  | 8582 | -0.184 | -0.1142 | Yes |
| 178 | NOP56 |  |  | 8619 | -0.199 | -0.1038 | Yes |
| 179 | LUC7L3 |  |  | 8631 | -0.203 | -0.0902 | Yes |
| 180 | PAICS |  |  | 8653 | -0.214 | -0.0770 | Yes |
| 181 | BRCA1 |  |  | 8659 | -0.217 | -0.0617 | Yes |
| 182 | PRDX4 |  |  | 8660 | -0.217 | -0.0458 | Yes |
| 183 | HELLS |  |  | 8720 | -0.260 | -0.0336 | Yes |
| 184 | CENPE |  |  | 8734 | -0.270 | -0.0154 | Yes |
| 185 | SMC4 |  |  | 8764 | -0.307 | 0.0037 | Yes |
Table: GSEA details [plain text format]

  

Fig 2: HALLMARK\_E2F\_TARGETS: Random ES distribution      
 Gene set null distribution of ES for **HALLMARK\_E2F\_TARGETS**
